# Personal

**Published:** 1909-08

**Authors:** 


### PERSONAL.

---

Dr. Ralph Robinson has been appointed Health Physician by the newly created City of Lackawanna, which joins Buffalo in our Southern border.

---

Dr. Henry Reed Hopkins, of Buffalo, formerly professor of hygiene, has been created Emeritus Professor of Hygiene at the University.

---

Dr. James W. Putnam, of 525 Delaware Avenue, Buffalo, one of the Associate Editors of the Buffalo Medical Journal, spends the summer in Europe. He sailed for Genoa via Naples and Gibraltar on July 17. Thence he goes to Venice and Innsbruck, where he will spend two weeks on a walking tour through the Tyrolean Alps. Later Paris will be visited and sometime spent at the Salpetriere and other neurological clinics. He sails from Cherbourg on August 29, returning to Buffalo early in September. Mrs. Putnam accompanies him. Meanwhile, Dr. L. Kauffman, his assistant, is taking care of his practice.

Dr. Max Breuer, of Buffalo, will sail on August 7, 1909, on the Kaiserin Augusta Victoria for a short visit in England. He will go from there to Germany to attend for a time the clinics of Berlin and Breslau. He will return in the autumn to resume his professional work at 33 Franklin Street.

---

Dr. Robert F. Sheehan, of this city, has been appointed lecturer on hygiene at the University of Buffalo.

---

Albert P. Sy, M. S., Ph.D., has received the appointment of professor of chemistry and toxicology in the medical department of the University of Buffalo.

---

Dr. Jane W. Carroll, Miss Rose Carroll, Miss Adele Carroll and Miss Emiline Carroll of Buffalo, sailed from New York, July 3, 1909, for a summer in Europe.

---

Dr. Regina Flood Keyes, of Buffalo, sailed the latter part of June, for Europe to spend the summer in visiting the hospitals.

---

Dr. Francis E. Fronczak, assistant health commissioner, represented the city at the convention of the National Education Association, recently held in Denver.

---

Dr. Ernest Wende, Health Commissioner of Buffalo, for the first time in several months was at his desk one day the latter part of June. He remained but a short time, being still weak from the long in-door confinement.

While at his desk, Dr. Wende announced the appointment of Lot H. Cooke as plumbing inspector in the health department. Mr. Cooke, who lives at No. 49 Buffam street, was first on the civil-service list. He will receive a salary of $1,200.

---

Dr. and Mrs. Willis G. Gregory, of Richmond avenue, Buffalo, are spending a few weeks in the Bermudas, on a visit to an old friend.

---

Dr. and Mrs. William Mansperger, of Main street, Buffalo, are making an extended Western trip, visiting the Yellowstone Park, the Seattle Exposition and Alaska, expecting to return about the first of September.

